# Prognostic value of HIF-1α in digestive system malignancies: evidence from a systematic review and meta-analysis 

**Published:** 2022

**Authors:** Mohammad-Hassan Arjmand, Ali Moradi, Hamid-Reza Rahimi, Ali Es-haghi, Abolfazl Akbari, Mohammad Reza Hadipanah, Jalil Afshar, Hassan Mehrad-Majd

**Affiliations:** 1 *Clinical Biochemistry Research Center, Basic Health Sciences Institute, Shahrekord University of Medical Sciences, Shahrekord, Iran*; 2 *Orthopedic Research Center, Mashhad University of Medical Sciences, Mashhad, Iran*; 3 *Department of Medical Genetics and Molecular Medicine, Faculty of Medicine, Mashhad University of Medical Sciences, Mashhad, Iran*; 4 *Department of Biology, Mashhad Branch, Islamic Azad University, Mashhad, Iran*; 5 *Colorectal Research Center, Iran University of Medical Sciences, Tehran, Iran*; 6 *Student Research Committee, Faculty of Medicine, Mashhad University of Medical Sciences, Mashhad, Iran *; 7 *Department of Biochemistry, Neyshabur Branch, Islamic Azad University, Neyshabur, Iran*; 8 *Cancer Molecular Pathology Research Center, Mashhad University of Medical Sciences, Mashhad, Iran*

**Keywords:** HIF-1α, Neoplasms, Digestive system, Prognosis

## Abstract

**Aim:**

This meta-analysis aimed to evaluate the association of HIF-1α expression with clinicopathological features and overall survival (OS) of patients with digestive system malignancies.

**Background:**

Numerous studies have demonstrated that hypoxia-inducible factor-1α (HIF-1α) is abnormally expressed in various solid tumors. However, the clinicopathological features and prognostic value of HIF-1α expression in patients with digestive system malignancies remain controversial.

**Methods:**

A literature search in PubMed, Web of Science, and Scopus databases was performed to identify all relevant studies published in English until 15 October 2020. The pooled effect was calculated to evaluate the association between HIF-1α expression and clinicopathological features and overall survival in cancer patients. Pooled odds ratios (ORs) or hazard ratios (HRs) with a 95% confidence interval (CI) were calculated using fixed- or random-effects model based on between-study heterogeneity.

**Results:**

A total of 44 eligible studies with 5,964 patients were included. The pooled results indicated a positive association of HIF-1α overexpression with poor overall survival (OS) (HR=1.990, 95% CI: 1.615-2.453, *p*<0.001) and disease-free survival (DFS) (HR=1.90, 95% CI: 1.084-3.329, *p*=0.043). Meta-analysis results showed that HIF-1α level expression was significantly associated with positive lymph node metastasis (OR=1.869, 95% CI: 1.488-2.248, *p*<0.001), distance metastasis (OR=2.604, 95% CI: 1.500-4.519, *p*<0.001), tumor stage (OR=1.801, 95% CI: 1.437-2.257, *p*<0.001) and tumor size (OR=1.392. 95% CI: 1.068-1.815, *p*=0.014).

**Conclusion:**

This meta-data suggest that HIF-1α expression might serve as an independent prognostic marker and a promising therapeutic target in patients with digestive system malignancies.

## Introduction

 The term “digestive system cancer” generally refers to those cancers that affect the gastrointestinal tract (GI tract) and accessory organs of digestion. This type of cancer is responsible for more cancer-related deaths than any other type (1, 2). The most commonly diagnosed digestive system malignancies include esophageal cancer, gastric cancer (GC), liver cancers, pancreatic cancer, and colorectal cancer (CRC) (3). They are reported to account for over 26% of newly diagnosed cases globally and 35% of all cancer-related deaths (4). Despite great advancements in cancer prevention and treatment during the past decades, the 5-year survival for patients with these malignancies has not been significantly improved. The lack of effective biomarkers as potential screening tools for early detection of cancer can be considered as a main reason. The identification of various prognostic and predictive biomarkers for patients with digestive system malignancies might provide to be essential information on the probability of response to a particular therapy.

Various factors including unhealthy lifestyle, genetic legions, comorbid conditions, and medications related to cancer treatment might affect digestive system malignancies in their different aspects such as progression, recurrence, and mortality (5, 6). Increasing the knowledge about the molecular mechanisms involved in these processes may lead to the identification of potential traditional protein- or genome-based markers with high predictive value for tumor behavior, then subsequent clinical management and optimal treatment of cancer patients (7). Hence, special efforts by researchers are required to identify clinically applicable biomarkers for patients affected with these kinds of cancer.

A common feature of most solid tumors, generated by abnormal microvasculature in rapidly proliferating tissues, is called hypoxia (8). Hypoxia promotes the expression of HIF-1α, a key transcription factor that regulates the expression of different genes related to various aspects of cancer biology, such as cell proliferation, angiogenesis, and glucose metabolism (8, 9). Moreover, in hypoxic conditions, intratumor cytokines, growth factors, and other signaling molecules stimulate HIF-1α expression and activity in tumor cells by different molecular mechanisms such as PI3K or MAPK (9). Mounting evidence has shown that HIF-1α activation induces cancer progression; hence, various clinical studies have demonstrated the association between overexpression of HIF-1α and mortality rates in many human cancer types (10, 11). Different studies have indicated the significant connection between high level expression of HIF-1α and poor OS and DSF in patients with digestive system malignancies such as esophageal cancer (EsoC) (12-14), colorectal cancer (CRC) (14-16), gastric cancer (GC) (17-19), pancreatic adenocarcinoma (PDAC) (20), and hepatocellular carcinoma (HCC) (10, 21, 22). However, to clarify to what extent HIF-1α expression level might be of prognostic significance in digestive system cancers, a comprehensive meta-analysis of previous studies is needed. Therefore, we conducted a systematic review of published studies to evaluate the potential prognostic value of HIF-1α expression in digestive system malignancies. 

## Methods

**Literature search procedures**

Searches were performed on the Web of Science, PubMed, Scopus, and Google Scholar to identify related studies in the English language published up to 15 October 2020. Search terms, used both individually and/or in various combinations, comprised HIF-1α, tumor, malignant, cancer, neoplasm, carcinoma, adenocarcinoma, hepatocellular, liver, gastric, stomach, gastro, esophageal, colon, colorectal, rectal, and pancreatic. Moreover, the references list in each selected article was checked to optimize sensitivity. 

**Selection criteria**

The current meta-analysis investigated the significance of HIF-1α in digestive system malignancies, including esophageal, gastric, liver, pancreas, and colorectal cancers. To meet the inclusion criteria for this research, the studies had to have: 1) provided pathological evidence to confirm digestive system malignancies, 2) examined the association between HIF-1α and clinic-pathological parameters of various types of digestive system cancers, 3) reported or provided data about disease-free survival (DFS) or overall survival (OS) rates, 4) evaluated HIF-1α expression in either tissue or serum/plasma, and 6) if studies included patients with different cancers, there must be a subgroup analysis of digestive system cancers. Articles were excluded if they: 1) focused on animals or cells to compare HIF-1α with non-human subjects, 2) were reviews, letters, case reports, editorials, or commentaries, 3) were duplicate publications, or 4) lacked key information to calculate hazard rations (HRs) with 95% confidence intervals (CIs). In the case of overlapping patients in more than one study, only the most complete study was enrolled. 

**Data extraction and methodological assessment**

The following information was extracted from each included study: first author’s surname, year of publication, country of origin, tumor type, tumor size, sample size, HIF-1α detection assay, tumor stage, lymph node metastasis (LNM), distance metastasis (DM), prognostic outcomes of interest, and HR with its 95% CI.

The quality of included studies was assessed independently by two authors (MHA and HMM) using the Newcastle-Ottawa scale (NOS) (23). All studies were scored (from 0 to 9) in terms of patient selection, study comparability, and outcome assessment. Any discrepancy was resolved by team consensus.

**Statistical analysis**

High and low HIF-1α expression rates were defined according to the arbitrary cut-off values provided by the literature. The odds ratios (ORs) and corresponding 95% CIs were applied to evaluate the association between HIF-1α expression and clinicopathological features. HRs in combination with the corresponding 95% CIs of identiﬁed studies were used to estimate the effect of HIF-1α expression on survival outcomes. HRs with 95% CIs were directly acquired from the articles or calculated indirectly using Kaplan–Meier curves according to the methods described by Parmar et al. (24), Williamson et al. (25), and Tierney et al. (26). As a rule, a pooled HR > 1 was assumed a poor prognosis for HIF-1α overexpression and considered statistically significant if the 95% CI did not cross one. Heterogeneity across the studies was quantified using the χ2-based Q test and I^2^ index. I^2^ > 50% or Q test *p* < 0.05 reflected significant heterogeneity across studies. In case of significant heterogeneity, the random effect model was adopted; otherwise, a fixed effect model was employed. Potential sources of heterogeneity were explored by performing subgroup, metaregression, sensitivity, and Galbraith plot analyses (27). Begg’s funnel plots and Egger’s linear regression test were also conducted to judge the probability of publication bias. All analyses were performed using the Comprehensive Meta-Analysis software. A *p*-value<0.05 was considered as statistically significant. 

## Results

**Literature search**

**Figure 1:**
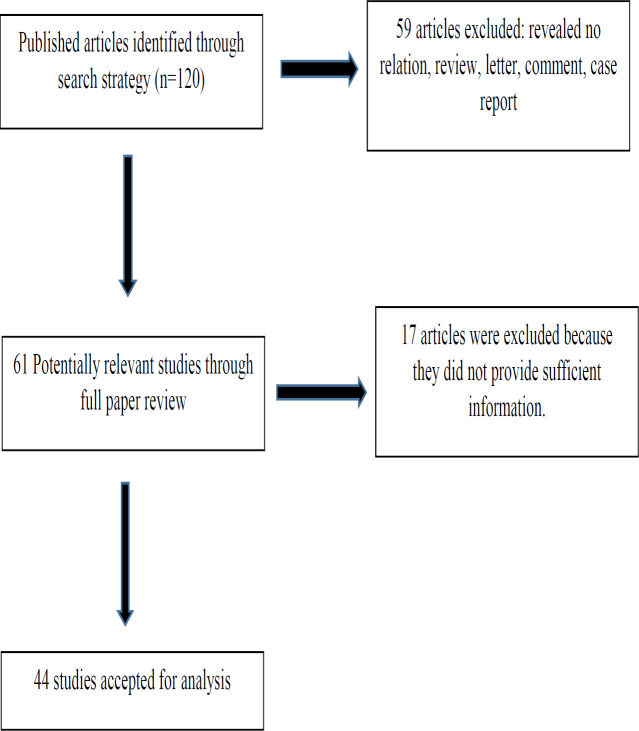
Flowchart of study selection process

A flow diagram of the study selection process is provided in Figure 1. In our initial searches, 120 potentially relevant articles were retrieved according to the predefined search strategy. In the first screening, 31 duplicate records were excluded, and in subsequent screening steps, 28 additional articles were excluded, because they were conference records, irrelevant to our topic, or non-original papers. A more detailed review resulted in the exclusion of another 17 studies due to insufficient information. Consequently, 44 eligible papers comprising 5,964 patients were included in the meta-analysis for quantitative analysis. The majority of studies were performed in Asia (17 from China, 15 from Japan, one from Korea, and one from Turkey); the remaining studies were from the UK, Germany, Greece, the USA, and Australia. 

**Study characteristics**

The general characteristics of the selected studies are summarized in Table 1. All studies were published between 2003 and 2019. The cancer types evaluated in this meta-analysis were: gastric cancer (n=13) (17, 19, 28-38), CRC (n=16) (16, 39-53), EsoC (n=9) (13, 14, 54-60), HCC (n=5) (10, 21, 61-63), and PDAC (n=1) (20). The studies investigated the association between HIF-1α expression and prognosis index, including OS and DFS. The cut off values for HIF-1α expression level varied throughout the studies. 

**Figure 2.**
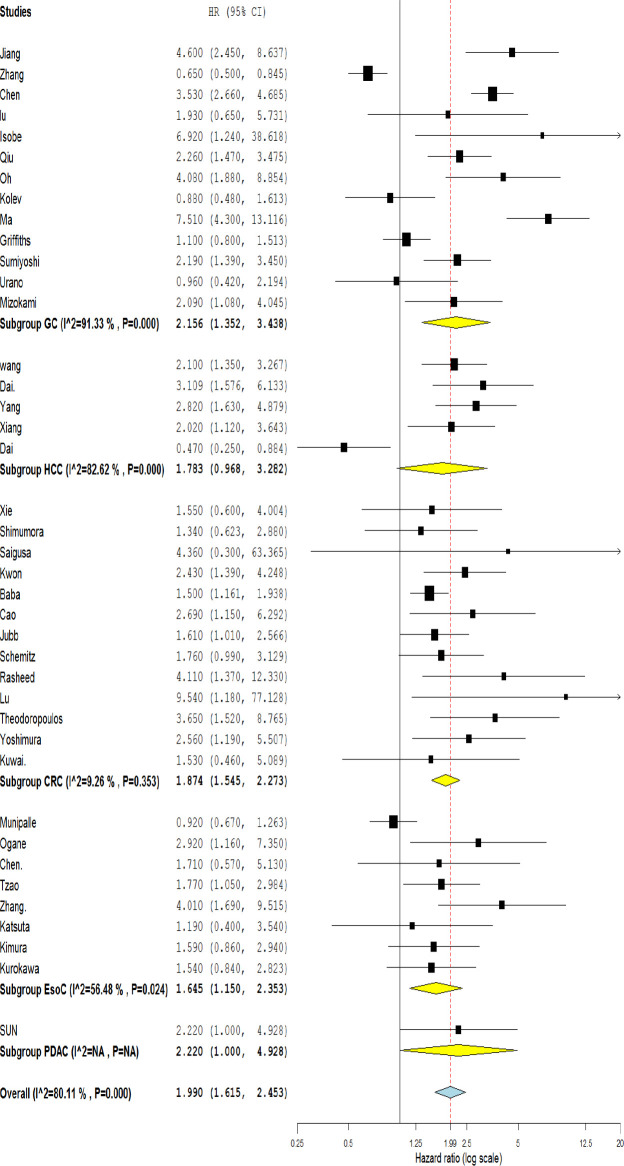
Forest plot showing the association between OS and HIF-1α expression in overall and based on different cancer types. (GC: Gastric Cancer, HCC: Hepatocellular carcinoma, CRC: Colorectal cancer, EsoC; Esophageal cancer, PDAC: Pancreatic ductal adenocarcinoma.)

Forty studies investigated the association of HIF-1α expression with OS, ten with DFS, thirty-four with lymph node metastasis (LNM), seventeen with distance metastasis (DM), twenty-nine with TNM stage, and nineteen studies with tumor size. According to the NOS scoring system, all included studies were awarded five or more stars and were considered as being of good quality.

**Figure 3:**
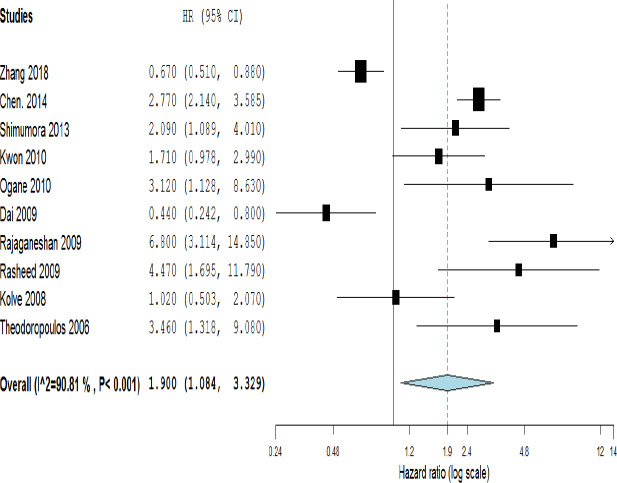
Forest plot showing the association between HIF-1α expression and DFS in different cancer types

**Figure 4:**
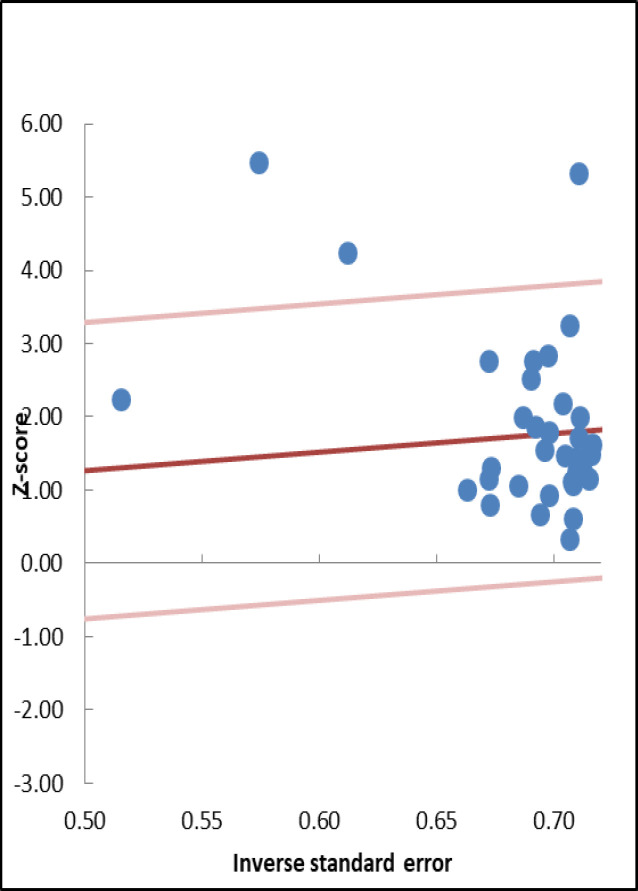
Galbraith plots of the association between HIF-1α expression and OS in different cancer types

**The relationship between HIF-1α expression and OS in digestive system cancers**

The data of forty eligible studies was summarized to assess the association between HIF-1α expression level and OS. Due to the significant heterogeneity of the reports (I^2^ = 80.11%, *p* < 0.001), a random effect model was applied to evaluate pooled HR. 

**Figure 5:**
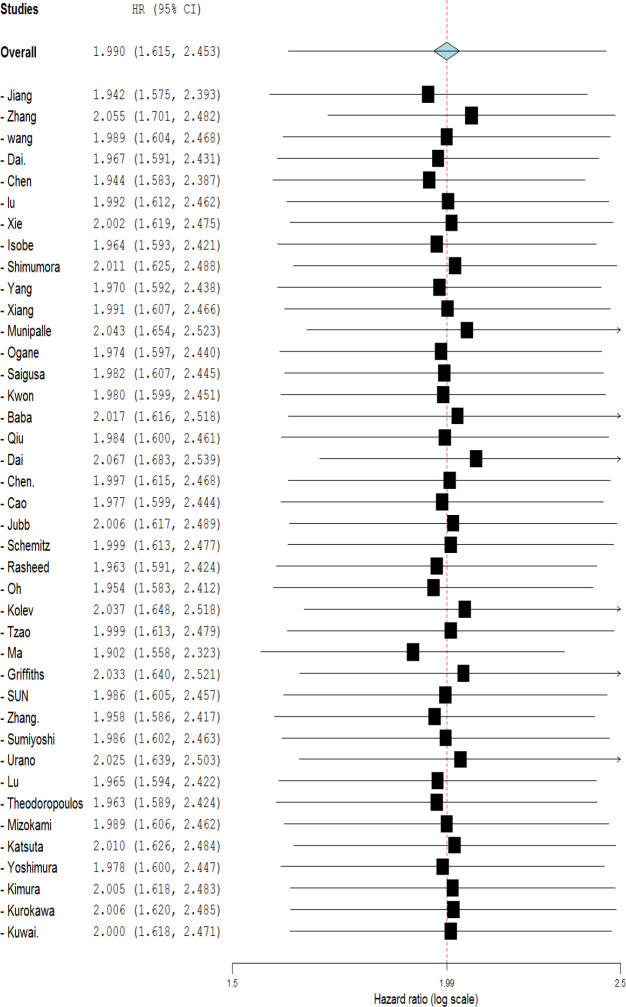
The sensitivity analysis for the meta-analysis of OS in tumor patients

Meta-results showed a significant association between the high expression of HIF-1α and poor OS (HR=1.990, 95% CI: 1.615-2.453, *p*<0.001) (Figure 2). Moreover, the combined data from ten studies reporting HR for DFS indicated a significant relationship between the high level of HIF-1α and poor outcome (HR=1.90, 95% CI: 1.08-3.33, *p*=0.043 and I^2^= 90.81% *p*<0.001) (Figure 3). To explore the source of heterogeneity, subgroup analyses were performed according to sample size, ethnicity, and cancer type. Subgroup analyses based on sample size revealed a significant correlation between high HIF-1α expression and patients’ overall survival in studies with both more and less than 100 cases (HR=1.918, 95% CI: 1.381-2.665, *p*=0.000; and HR=2.008, 95% CI: 1.578-2.554, *p*=0.001; respectively) (Table 2). Ethnicity-based subgroup analysis also indicated a significant association between the expression of HIF-1α and poor OS in Asians (HR=2.010, 95% CI: 1.590-2.541, *p*=0.000) and Caucasians (HR=1.854, 95% CI: 1.171-2.936, *p*=0.008) (Table 2). According to subgroup analysis based on cancer type, there was a significant association between HIF-1α expression and poor OS in GC (HR=2.156, 95% CI: 1.352-3.438, *p*<0.000), CRC (HR=1.874, 95% CI: 1.545-2.273, *p*<0.001), and EsoC (HR=1.645, 95% CI: 1.150-2.353, *p*=0.024); however, no significant association was seen regarding the HCC (HR=1.783, 95% CI: 0.968-3.282, *p*=0.063) (Figure 2). Meta‐regression was performed to find any evidence of covariates affecting OS. The results showed that neither sample size, ethnicity, nor cancer type, alone or in combination, significantly affected OS (Table 2).

**Figure 6:**
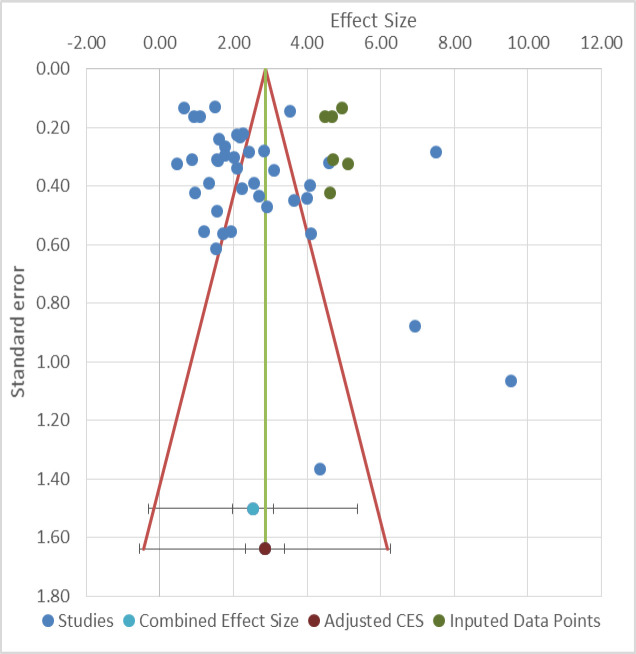
Funnel plot analysis of potential publication bias for meta-analysis

**Association of HIF-1α expression with clinicopathological characteristics**

A meta-analysis was performed to assess the correlation between HIF-1α expression level and tumor clinicopathological characteristics. The pooled ORs and 95% CIs of all characteristics including tumor size, stage of tumor, LNM, and DM as well as age and sex are presented in Table 3. High expression of HIF-1α showed a significant association with tumor size (OR=1.392. 95% CI: 1.068-1.815, *p*=0.014, Random effect), stage (OR=1.801, 95% CI: 1.437-2.257, *p*<0.001, Random effect), LNM (OR=1.869, 95% CI: 1.488-2.248, *p*<0.001, Random effect), and DM (OR=2.604, 95% CI: 1.500-4.519, *p*<0.001, Random effect). However, no significant difference was observed between HIF-1α expression and age (OR=0.925, 95% CI: 0.777-1.101, *p*=0.3, Random effect) or sex (OR=1.69, 95% CI: 0.822-1.391, *p*=0.659, Random effect).

**Table 1:** Basic characteristics of the included studies

Author/ year	Age (High/ Low)		Country	Tumor type	Tumor size (High/Low)	Sample size	Male (High/Low) Female (High/Low)	TNM Stage (High/Low)	HIF-1α Expression						Survival analysis	Hr (95% CI)		Method		Sample type	NOS score
									High			Low									
									Total	LNM	DM	Total	LNM	DM							
Jiang 2019	≥61(39-24) <61(43-18)		China	GC	≥6cm (50/33) <6cm(32/9)	124	63/33 19/9	I/II (28/24) III/IV(54/18)	82	59	-	42	24	-	OS	4.60 (2.45-8.66)		IHC/ RT PCR		T	8
Zhang 2018	≥60(113/116) <60(106/93)		China	GC	≥5cm (100/85) <5cm(119/124)	428	155/149 64/60	I/II(95/119) III(144/90)	219	151	32	209	98	14	OS/ DFS	OS: 0.65(0.50-0.85) DFS:0.67(0.52-0.88)		IHC		T	8
Wang 2018	≥55(87/89) <55(136/101)		China	HCC	≥5cm (89/52) <5cm(126/133)	419	157/66 151/39	I (91/98) II/III (132/92)	223	-	-	190	-	-	OS	2.10 (1.35-3.26)		IHC		T	8
Dai 2018	≥50(23/40) <50(16/11)		China	HCC	≥5cm(19/21) <5cm(19/30)	90	35/49 4/2	-	39	-	-	51	-	-	OS	3.109 (1.576-6.131)		IHC		T	8
Saka 2017	≥50(68/65) <50(23/30)		Turkey	CRC	≥5cm (44/55) <5cm(47/40)	186	57/43 34/52	I/II (29/33) III/IV (62/62)	91	53	44	95	56	52	OS	-		IHC		T	8
Chen 2014	≥60(117/105) <60(100/124)		China	GC	≥5cm (122/131) <5cm (95/98)	446	172/176 45/53	I/II(61/71) III/IV(156/158)	217	173	63	229	176	25	OS/ DFS	OS: 3.53(2.66-4.66) DFS: 2.77(2.14-2.6)		IHC		T	8
Lu 2013	≥49.82(25/18) <49.82(11/14)		China	GC	≤5cm (9/15) >5cm (27/17)	68	21/22 15/10	I/II(10/16) III/IV(26/16)	36	27	9	32	20	6	*OS	1.93 (0.65-5.75)		IHC		T	8
Yang 2013	≥50(52/40) <50(20/14)		China	HCC	≥5cm(40/26) <5cm(32/28)	126	5/105 2/14	NR	72	-	-	54	-	-	OS/DFS	2.82 (1.63-4.90)		IHC		T	8
Shimomura 2013	≥61(10/25) ≤60(10/19)		Japan	CRC	≥3cm(10/22) <3cm(13/32)	64	11/31 9/13	16/25	20	-	-	44	-	-	OS/DFS	OS: 1.34 (0.62-2.88) DFS: 2.09 (1.09-4)		IHC		T	8
Xie 2013	≤60(12/17) >60(16/15)		China	CRC	≤2cm (6/18) >2cm (22/14)	60	21/19 7/13	I/II(13/13) III/IV(15/19)	28	16	-	32	11	-	OS	1.550.60-4.01)		IHC		T	8
Isobe 2013	≥65(48/30) <65(36/14)		Japan	GC	NR	128	56/35 28/9	NR	84	50	20	44	12	2	OS	6.92(1.24-130.4)		IHC		T	8
Xiang 2012	≤50(19/17) >50(11/22)		China	HCC	≤5cm (18/15) >5cm (12/24)	69	27/34 3/5	I/II(15/23) III/IV(15/16)	30	21	-	39	23	-	OS	2.02 (1.12-3.66)		IHC		T	8
Ogawa 2011	≥60(7/7) <60(4/4)		Japan	EsoC	NR	37	10/14 1/0	NR	11	9	4	14	8	1	RFS	0.071 (0.015-0.34)		IHC		T	8
Shioya 2011	NR		Japan	CRC	NR	50	38	NR	21	17	-	29	18	-	RFS	4.13 (1.52-11.24)		IHC		T	8
Munipalle 2011	>70(13/5) <70(6/12)		UK	EsoCl	NR	36	8/7 11/10	I/II (4/3) III/IV (17/12)	19	14	8	17	13	4	OS	0.92 (0.67-1.31)		IHC		T	8
Ogane 2010	≤62(15/31) >62(34/16)		Japan	EsoC	NR	96	60/29 5/2	NR	65	35	-	31	9	-	OS/DFS	OS:2.92(1.16-7.32) DFS:3.12(1.28-8.63)		IHC		T	8
Saigusa 2010	≥65		Japan	CRC	NR	52	42/10	NR	-	-	-	-	-	-	OS	OS : 4.36(0.05-0.91)		IHC/RT-PCR		T	8
Kwon 2010	-		Korea	CRC	≥5cm (42/56) <5cm (21/29)	311	109/62 87/53	I/II (100/73) III/IV (96/42)	63	-	-	85	-	-	*OS/DFS	OS:2.43(1.39-4.21) DFS:1.71(0.98-2.99)		IHC		T	8
Baba 2010	≥70(53/223) <70(89/366)		USA	CRC	NR	731	41/220 101/369	I/II (42/180) III/IV (74/234)	142	-	-	589	-	-	OS	1.50 (1.16-1.94)		IHC		T	8
Qiu 2010	≤60(58/41) >60(52/37)		China	GC	≤5cm (36/52) >5cm (74/23)	188	72/55 38/23	I/II(41/56) III/IV(69/22)	110	82	-	74	44	-	OS	2.26(1.47-3.48)		IHC		T	8
Dai 2009	≤52(26/28) >52(16/40)		China	HCC	≤5cn (21/35) >5cm (28/38)	110	38/57 4/11	I/II(18/40) III/IV(24/28)	42	-	-	68	-	-	OS/DFS	OS: 0.47(0.25-0.89) DFS: 0.44(0.25- 0.8)		IHC/ RT-PCR		T	8
Chen 2009	<60(17/13) ≥60(8/16)		China	EsoC	NR	54	15/23 10/6	I/II(5/17) III/IV(20/12)	25	16	-	29	12	-	*OS	1.71(0.57-5.13)		IHC		T	8
Cao 2009	≥60(24/23) <60(15/9)		China	CRC	≥5cm (22/18) <5cm (1714)	71	24/19 15/13	I/II(15/27) III/IV(24/5)	39	22	10	32	5	1	OS	2.69(1.15-6.30)		IHC		T	8
Jubb 2009	NR		Australia	CRC	NR	164	NR	NR	95	-	-	60	-	-	OS	1.61(1.01-2.57)		IHC		T	8
Rajaganesh2009	NR		UK	CRC	NR	55	NR	NR	25	-	-	30	-	-	*DFS	6.8(3.11-14.85		IHC		T	8
Schemitz 2009	NR		German	CRC	NR	135	NR	I/II (9/38) III/IV(24/48)	34	22	13	90	43	16	OS	1.76(0.99-3.22)		IHC		T	8
Rasheed 2009	NR		UK	CRC	NR	NR	32/24 16/18	I/II(258/32) III/IV(23/10)	48	23	-	42	10	-	OS/DFS	4.11(1.37-12.35) 4.47 (1.68-11.89)		IHC		T	8
Oh 2008	≥60(9/47) <60(9/49)		Korea	GC	≥4cm (14/53) <4cm (4/43)	114	10/57 8/39	I/II(74/60) III/IV(11/34)	18	15	-	96	58	-	OS	4.08(1.88-8.88)		IHC		T	8
Kolev 2008	≥60(49/26) <60(46/31)		Japan	GC	≥5cm (48/24) <5cm (47/33)	152	72/38 23/19	I/II(62/42) III/IV(35/15)	95	50	-	57	21	-	OS/ DFS	0.88(0.48-1.62) 1.02(0.5-2.07)		IHC		T	8
Tzao 2008	≤70(36/24) >70(16/9)		China	EsoC	NR	85	47/33 5/0	I/II(25/23) III/IV(27/10)	52	29	13	33	12	4	OS	1.77(1.05-2.97)		IHC		T	8
Ma 2007	NR		China	GC	NR	118	NR	NR	58	50	47	60	33	8	OS	7.51(4.30-13.11)		IHC		T	8
Griffiths 2007	NR		UK	GC	NR	80	NR	I/II(41/44) III/IV(51/37)	93	64	2	83	60	2	OS	1.10(0.8-1.4)		IHC		T	8
SUN 2007	≥60(19/16) <60(7/16)		USA	PDAC	≥2cm (25/25) <2cm (1/7)	58	16/21 10/11	I/II(10/26) III/IV(16/6)	26	12	-	32	6	-	OS	2.22(1.00-4.99)		IHC		T	8
Sumiyoshi 2006	NR		Japan	GC	NR	216	56/92 29/39	I/II (45/80) III/IV(40/60)	85	49	5	131	61	4	OS	2.19(1.39-3.47)		IHC		T	8
Zhang 2007	>63(18/8) ≤63(16/10)		China	EsoC	NR	50	27/10 7/6	NR	34	31	7	16	10	0	*OS	4.01(1.69-9.51)		IHC		T	8
Urano 2006	NR		Japan	GC	NR	146	NR	I/II(46/25) III/IV(43/32)	83	36	-	55	28	-	*OS	0.96(0.42-2.20)		IHC		T	8
Lu 2006	NR		China	CRC	NR	30	NR	NR	19	12	-	11	0	-	*OS	9.54(1.18-70.66)		IHC		T	8
Theodoropoulos 2006	≤68(24/20) >68(20/28)		Greece	CRC	>3cm (36/39) <3cm (8/9)	92	24/31 20/17	NR	44	30	-	48	21	-	OS/DFS	OS:3.65(1.52-8.81) DFS: 3.46(1.32-9.8)		IHC		Tissue	8
Mizokami 2006	≥65(20/46) <65(29/31)		Japan	GC	≥3cm (39/42) <3cm (10/35)	126	35/50 14/27	NR	49	25	-	77	26	-	OS	2.09 (1.08-4.06)		IHC		Tissue	8
Katsuta 2005	NR		Japan	EsoC	NR	48	24/11 10/3	I (16/9) II/III/IV(18/5)	34	15	4	14	2	1	*OS	1.19(0.4-5.39)		IHC / RT-PCR		Tissue	8
Yoshimura 2004	NR		Japan	CRC	NR	NR	19/32 20/16	I/II(12/16) III/IV(27/32)	39	14	-	48	20	-	*OS	2.56(1.19-5.50)		IHC		Tissue	8
Kimura 2004	≤60(10/19) ≥61(22/31)		Japan	EsoC	NR	82	31/41 1/9	I/II(9/21) III/IV(23/29)	32	20	-	50	30	-	*OS	1.59(0.86-2.97)		IHC/ RT.PCR		Tissue	7
Kurokawa 2003	<60(26/18) ≥60(64/22)		Japan	EsoC	>4.5cm (47/23) <4.5cm (43/17)	130	79/34 11/6	I/II(64/17) III/IV(26/23)	90	38	11	40	28	11	OS	1.54(0.84-2.84)		IHC		Tissue	8
Kuwai 2003	NR		Japan	CRC	≥5cm (39/21) <5cm (42/37)	139	NR	NR	81	46	21	58	24	5	*OS	1.53 (0.46-5.13)		IHC		Tissue	7

Abbreviations: Hr: Hazard ratio, CI: Confidence interval, NOS: Newcastle-Ottawa Scale, UK: united kingdom, USA, Unites states of America, GC: Gastric Cancer, HCC: Hepatocellular carcinoma, CRC: Colorectal cancer, EsoC; Esophageal cancer, PDAC: Pancreatic ductal adenocarcinoma, OS: overall survival, DFS: Disease free survival, LNM: lymph node metastasis, DM: distant metastasis. IHC: immunohistochemistry, RT-PCR: real time-PCR, T: Tissue, NR: not reported, Note: The dashes represent no data.

**Table 2:** Stratified analyses of pooled hazard ratios for overall survival

Stratified analysis		No. of studies	No. of patients	Test of association		Test of heterogeneity			P- Value^b^
				Pooled HR (95% CI)	p-value	I^2^ (%)	P-value^a^	Model	
Overall survival (OS)		40	5636	1.99 (1.62-2.45)	<0.001	80.11	<0.001	R	-
Disease free survival (DFS)		10	1970	1.90 (1.08-3.33)	0.043	90.81	<0.001	R	-
Sample size	>100	19	4637	1.92 (1.38- 2.66)	<0.001	88.4	<0.001	R	0.685
	<100	21	1327	2.01 (1.58- 2.55)	<0.001	55.7	<0.001	R	
Ethnicity	Asian	36	4613	2.01 (1.59- 2.54)	<0.001	81.17	<0.001	R	0.818
	Caucasian	6	1351	1.85 (1.17- 2.94)	0.008	71.03	0.004	R	
Cancer type	CRC	13	2140	1.94 (1.58-2.39)	<0.001	9.26	0.325	F	0.910
	EsoC	8	618	1.64 (1.15-2.35)	0.006	56.48	0.024	R	
	GC	13	2334	2.16 (1.35-3.44)	<0.001	91.33	<0.001	R	
	HCC	5	814	1.78 (0.97-3.28)	0.063	87.61	<0.001	R	
	PDAC	1	58	-	-	-	-	-	

^a^ P-Value for heterogeneity within each subgroup. ^b ^P-Value for heterogeneity between subgroups with meta-regression analysis CRC: Colorectal cancer, EsoC; Esophageal cancer, GC: Gastric Cancer, HCC: Hepatocellular carcinoma, PDAC: Pancreatic ductal adenocarcinoma, R: random effect model, F: fixed effects model

**Table 3:** Meta-analysis of the association between HIF-1α expression and clinicopathological characteristics

Stratified analysis	No. of studies	No. of patients	Test of association		Test of heterogeneity		
			Pooled OR (95% CI)	p-value	I^2^ (%)	P-value	Model
Gender (male vs. female)	32	4995	0.94 (0.70-1.25)	0.659	72.72	<0.001	R
Age (≥55 vs. <55)	28	4472	0.93 (0.78-1.10)	0.382	35.9	0.03	R
Tumor size (large vs. small)	21	3571	1.39 (1.07-1.82)	0.022	68.65	<0.001	R
LNM (yes vs. no)	33	3822	1.87 (1.49-2.35)	<0.001	58.62	<0.001	R
DM(yes vs. no)	16	2353	2.60 (1.50-4.52)	<0.001	76.38	<0.001	R
Tumor stage (III+IV vs. I+II)	28	4462	1.80 (1.44-2.26)	<0.001	61.65	<0.001	R

**Sensitivity analysis and publication bias**

Galbraith plot detected three studies as the outliers with possible contributions to heterogeneity (Figure 4) (17, 29, 30). Sensitivity analysis was done to evaluate the robustness of the results. No single study was found to significantly change the direction of the HRs and ORs (Figure 5). Begg's funnel plot and Egger's test revealed a significant publication bias across the included studies (Egger's test, *p*=0.024) (Figure 6).

## Discussion

The current meta-results indicate that high expression of HIF-1α is associated with poor prognosis in patients with digestive system malignancies. Subgroup analysis with regard to cancer type showed a positive correlation between HIF-1α expression and poor OS in EsoC, GC, and CRC. However, no correlation was observed between HIF-1α expression and poor OS in HCC. More studies are needed to elucidate the role of HIF-1α in HCC. Additionally, subgroup analyses according to ethnicity and sample size showed that HIF-1α expression was related to worse OS. Elevated HIF-1α expression was also positively associated with four clinicopathological characteristics, namely LNM, DM, tumor size, and clinical stage of tumor. This could confirm the fact that HIF-1α overexpression plays a critical role in the biological behavior of different solid tumors. Pooled data demonstrated that high HIF-1α expression can act as a significant prognostic factor for survival outcomes and can provide a new reference point for predicting the metastasis and progression of cancer. 

It is well known that the HIF-1α transcription factor upregulates and promotes the expression of many genes that are critical for cellular function (64). A possible explanation for this strong relationship between HIF-1α overexpression and tumor clinicopathologic factors could be the direct regulatory effect of HIF-1α on the vascular endothelial growth factor (VEGF) gene which is responsible for tumor angiogenesis (65). Angiogenesis is essential for the process of solid tumor formation, invasion, and metastatic spread. Moreover, HIF-1α may play a central role in tumorigenesis by upregulating different signaling pathways such as Myc and PI3K/AKT/mTOR that are involved in tumor proliferation, differentiation, migration, and invasion (66). Recent studies have confirmed that the overexpression of HIF-1α is associated with the aggressive phenotype of tumors. 

According to the current results and in line with those of other studies, the relationship between HIF-1α expression and worse outcomes suggest HIF-1α as a target for therapeutic uses. HIF-1α target therapy may increase the survival of patients with advanced GI malignancies undergoing chemotherapy or radiotherapy. The data further suggests an important role for HIF-1α in GI cancer progression and poor OS in Asians and Caucasians. Moreover, HIF-1α expression is related to poor OS in both genders; hence, it may be a potential therapeutic target for cancer stratification in both genders. 

The current meta-analysis had some limitations. First, all included studies were published in English, which may be a source of limited generalizability and selection bias. Second, the considerable heterogeneity might affect the study results. However, to minimize the effect of heterogeneity, a random effect model was applied. Third, HRs in a few of the selected studies were extracted from the Kaplan-Meier curve, which might not reflect true values. Finally, there is no standard threshold or definite cut-off value for HIF-1α expression in digestive system malignancies. 

The current meta-analysis indicates that overexpression of HIF-1α is associated with poor prognosis in patients with digestive system malignancies and might be a novel prognostic factor for patient survival. The data also demonstrates that elevated HIF-1α is correlated with clinicopathological features such as LNM, DM, advanced TNM stage, and larger tumor size in digestive system cancers. HIF-1α has the potential to serve as a tumor marker for predicting the prognosis of digestive system malignancies.

## Conflict of interests

The authors declare that they have no conflict of interest.
